# dbCRY: a Web-based comparative and evolutionary genomics platform for blue-light receptors

**DOI:** 10.1093/database/bau037

**Published:** 2014-05-09

**Authors:** Yong-Min Kim, Jaeyoung Choi, Hye-Young Lee, Gir-Won Lee, Yong-Hwan Lee, Doil Choi

**Affiliations:** ^1^Department of Plant Science and Plant Genomics and Breeding Institute, Seoul National University, Seoul 151-921, Korea, ^2^Department of Agricultural Biotechnology, Fungal Bioinformatics Laboratory, Seoul National University, Seoul 151-921, Korea, ^3^Department of Bioinformatics and Life Science, Soongsil University, Seoul 156-743, Korea and ^4^Center for Fungal Genetic Resources and Center for Fungal Pathogenesis, Seoul National University, Seoul 151-742, Korea

## Abstract

Cryptochromes are flavoproteins that play a central role in the circadian oscillations of all living organisms except archaea. Cryptochromes are clustered into three subfamilies: plant-type cryptochromes, animal-type cryptochromes and cryptochrome-DASH proteins. These subfamilies are composed of photolyase/cryptochrome superfamily with 6–4 photolyase and cyclobutane pyrimidine dimer photolyase. Cryptochromes have conserved domain architectures with two distinct domains, an N-terminal photolyase-related domain and a C-terminal domain. Although the molecular function and domain architecture of cryptochromes are conserved, their molecular mechanisms differ between plants and animals. Thus, cryptochromes are one of the best candidates for comparative and evolutionary studies. Here, we have developed a Web-based platform for comparative and evolutionary studies of cryptochromes, dbCRY (http://www.dbcryptochrome.org/). A pipeline built upon the consensus domain profile was applied to 1438 genomes and identified 1309 genes. To support comparative and evolutionary genomics studies, the Web interface provides diverse functions such as (i) browsing by species, (ii) protein domain analysis, (iii) multiple sequence alignment, (iv) homology search and (v) extended analysis opportunities through the implementation of ‘Favorite Browser’ powered by the Comparative Fungal Genomics Platform 2.0 (CFGP 2.0; http://cfgp.snu.ac.kr/). dbCRY would serve as a standardized and systematic solution for cryptochrome genomics studies.

**Database URL:** http://www.dbcryptochrome.org/

## Introduction

Light is one of the major sources of energy and provides a signal for all living organisms from single cells to multicellular organisms. Light perception and light-mediated signaling, which are important for growth and development as well as survival and adaptation, are achieved by a single or various combinations of photoreceptors. Among light responses, circadian rhythm is mediated by cryptochromes. Cryptochromes are flavoproteins that share a structural similarity to DNA photolyases and act as blue-/ultraviolet A (UV-A)-light receptors (1). Cryptochromes are widely distributed in bacteria and eukaryotes but are not found in archaea. Although initially characterized in plants (2), cryptochromes have also been found in animals including fruit fly (3), frog (4), chicken (5) and human (6–8).

Cryptochromes can be clustered into three subfamilies based on their sequence similarities: plant-type cryptochromes, animal-type cryptochromes and cryptochrome-DASH (CRY-DASH) proteins (9), and these subfamilies are composed of photolyases/cryptochromes superfamily with 6–4 photolyase and cyclobutane pyrimidine dimer (CPD) photolyase. Cryptochromes have two domains—an N-terminal photolyase-related (PHR) domain and a C-terminal domain of varying size for nuclear/cytosol trafficking and protein–protein interactions. The C-terminal domain is less conserved than the PHR domain, and the C-terminal domain of the plant-type cryptochromes is often longer than that of the animal-type cryptochromes. CRY-DASH proteins lack the C-terminal domain. Animal-type cryptochromes are a component of the circadian clock, which controls daily physiological and behavioral rhythms. Plant-type cryptochromes, however, mediate various photomorphogenetic responses such as the inhibition of stem elongation, the stimulation of leaf expansion, the control of photoperiodic flowering and regulation of gene expression in addition to the entrainment of the circadian clock (10, 11).

Although the molecular function of the central circadian oscillator is similar between animal-type and plant-type cryptochromes, the molecular mode of action between these two cryptochrome subfamilies are different. In animals, the transcription of time genes is regulated by a physical interaction of promoter-binding transcription regulators with cryptochromes; no such interaction has been found in plant-type cryptochromes. A previous phylogenetic study has suggested that the ancestral cryptochromes may have evolved before the divergence of prokaryotes and eukaryotes (12). In addition, the presence of DQXVP-acidic-STAES (DAS) sequences in cryptochromes suggests that the evolution of cryptochromes in plants occurred >400 million years ago even before the wide spread of vascular plants (1). These evolutionary studies also suggest that the divergence of cryptochromes from photolyases occurred earlier than the divergence of monocots and eudicots, and that the evolution of cryptochromes may have occurred in a lineage-specific and an environment-dependent manner (13). Thus, the molecular function and sequence similarities of cryptochromes are more diverse in each lineage compared with other photoreceptors such as phytochromes.

In plants, two approaches have been used to investigate the structure–function relationship of cryptochromes. One approach has investigated the mutation of individual amino acids in the cryptochrome. The other approach has observed the *in planta* behavior of a recombinant protein that contains a marker enzyme fused to a partial or full-length cryptochrome sequence. These studies have indicated that no specific region of cryptochromes has accumulated mutations with a significantly high frequency, but these mutations have accumulated with a relatively higher frequency in the 160-residue region of the C-terminal domain of phytochromes (9, 14). Although recent studies of cryptochromes have made considerable progress in the investigation of their molecular mechanisms and protein structures, there are still many intriguing open questions.

Recently, genome sequences from various organisms have been extensively analyzed, and accumulated DNA sequence information provides an opportunity to perform comparative studies on genes of interest. However, a flood of information prevents researchers from accessing and analyzing all of the available data. Thus, the construction of a database of individual gene families has become a requirement for enhancing data accessibility. In this article, we present dbCRY, a cryptochrome database. dbCRY is designed to serve as a central platform for providing and collecting information about cryptochromes from a wide range of organisms. dbCRY also provides analysis tools for detailed cryptochrome research.

## Construction and Evaluation of Data Analysis

**Figure 1.**
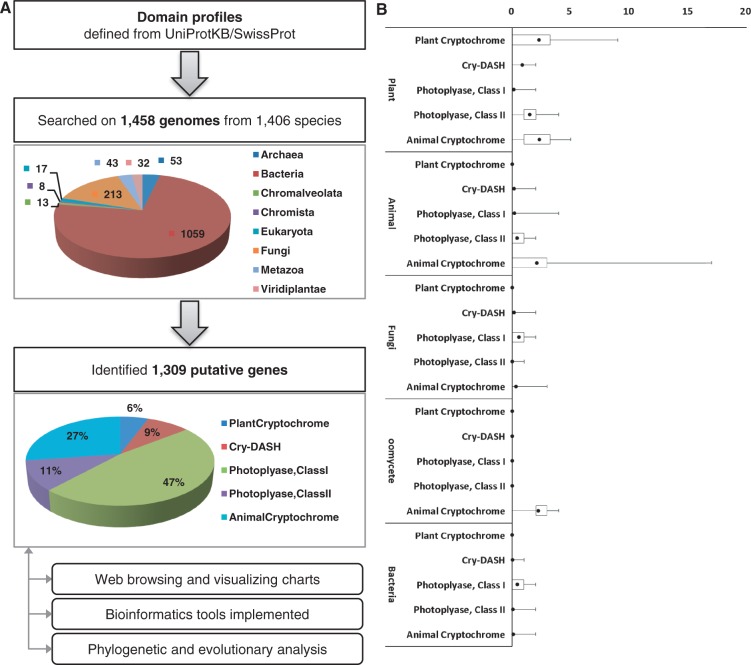
(**A**) The pipeline for the identification of cryptochrome-encoding genes. To construct the pipeline, expert-curated protein sequences annotated as cryptochrome or DNA photolyase were collected from UniprotKB/SwissProt and analyzed by InterPro scan. Cryptochrome genes were identified from 1458 genomes belonging to 1406 species and classified into five subfamilies. The identified 1309 genes were archived in the database. (**B**) The box plot represents a normalized number of cryptochrome/photolyase genes per genome.

To identify putative genes that encode photolyases or cryptochromes, five subfamilies were defined based on previous research (9, 15), and protein domain profiling was performed (Table 1). To determine the domain profiles for each class, all protein sequences from UniprotKB/SwissProt (16) that are annotated as DNA photolyase, cryptochrome or CRY-DASH proteins were retrieved. Retrieved proteins were turned out to have three essential domains for cryptochromes, which include IPR005101 [DNA photolyase, flavin adenine dinucleotide (FAD)-binding/cryptochrome, C-terminal], IPR006050 (DNA photolyase, N-terminal) and IPR014729 (Rossmann-like alpha/beta/alpha sandwich fold). To identify putative genes that encode cryptochromes, >7.5 million protein sequences from 1438 genomes in bacteria, archaea, oomycetes, fungi, plants and animals were screened. A total of 1309 protein sequences that had all three essential cryptochrome domains were identified. By identifying the presence and absence of additional domains, these 1309 protein sequences were further classified into five subfamilies: animal-type cryptochromes, CRY-DASH proteins, CPD photolyase class I, CPD photolyase class II and plant-type cryptochromes (Figure 1).

**Table 1.** InterPro domain profile for identification of cryptochromes

Class name	IPR number	Description
Plant-type cryptochrome	IPR014134	Cryptochrome, plant
	IPR014729	Rossmann-like alpha/beta/alpha sandwich fold
	IPR006050	DNA photolyase, N-terminal
	IPR005101	DNA photolyase, FAD-binding/cryptochrome, C-terminal
	IPR002081	Cryptochrome/DNA photolyase, class 1
	IPR018394	Cryptochrome/DNA photolyase, class 1 conserved site, C-terminal
CRY-DASH	IPR014133	Cryptochrome, DASH
	IPR014729	Rossmann-like alpha/beta/alpha sandwich fold
	IPR006050	DNA photolyase, N-terminal
	IPR005101	DNA photolyase, FAD-binding/cryptochrome, C-terminal
	IPR002081	Cryptochrome/DNA photolyase, class 1
CPD photolyase, class I	IPR002081	Cryptochrome/DNA photolyase, class 1
	IPR018394	Cryptochrome/DNA photolyase, class 1 conserved site, C-terminal
	IPR014729	Rossmann-like alpha/beta/alpha sandwich fold
	IPR006050	DNA photolyase, N-terminal
	IPR005101	DNA photolyase, FAD-binding/cryptochrome, C-terminal
CPD photolyase, class II	IPR008148	DNA photolyase, class 2
	IPR014729	Rossmann-like alpha/beta/alpha sandwich fold
	IPR006050	DNA photolyase, N-terminal
	IPR005101	DNA photolyase, FAD-binding/cryptochrome, C-terminal
Animal-type cryptochrome	IPR014729	Rossmann-like alpha/beta/alpha sandwich fold
	IPR006050	DNA photolyase, N-terminal
	IPR005101	DNA photolyase, FAD-binding/cryptochrome, C-terminal

A total of 106 protein sequences that are known to belong to photolyase/cryptochrome superfamily were collected from a literature survey to evaluate robustness of the identification pipeline (9, 15). The sequences were subjected to be searched by InterPro scan to obtain domain profiles. As a result, 98.11% of tested characterized sequences (104 of 106) were correctly classified by our domain profiles for each corresponding class, which demonstrate the accuracy and robustness of our pipeline (Table 2). In particular, 23 CRY-DASH, 8 CPD photolyase class I and 17 CPD photolyase class II genes were detected by the pipeline. In addition, 94.12 and 97.00% of genes encoding plant-type and animal-type cryptochrome proteins, respectively, were positively identified. Only two sequences in the test set showed prediction different from the reality. The plant-type cryptochrome, Q84L83 (*Armoracia rusticana*), was negatively predicted because it lacked the IPR002081 domain. The amino acid alignment of this protein with the CRY2 gene from *Arabidopsis thaliana,* a closely related species of *A.* *rusticana,* indicated there might be an annotation error in the C-terminal of this protein. The animal-type cryptochrome, Q17DK5 (*Aedes aegypti*), was assigned to CPD photolyase class I for having additional domains (IPR002081 and IPR018394).

**Table 2.** Identification and assignment of genes reported as a photolyase/cryptochrome gene family in previous studies

Validation result	Plant-type cryptochromes	CRY-DASH	CPD I	CPD II	Animal-type cryptochromes	Sum
Positive prediction	17	23	8	17	39	104
Negative prediction	1	0	0	0	1	2

The cryptochromes identified and assigned to five subfamilies in previous studies were tested to validate accuracy of in-house pipeline.

CPD I, CPD photolyase class I; CPD II, CPD photolyase class II.

High variability was observed in the length of full-length genes and core domains of plant- and animal-type cryptochromes. In addition, plant- and animal-type cryptochromes had a C-terminal extension (CCE) domain on their C-terminal end, and these domains showed a high variability in length. Furthermore, the domain architecture of animal-type cryptochromes was relatively simple compared with other subfamilies. Animal-type cryptochromes had only essential domains, whereas other subfamilies had additional domains (Table 1). So animal-type cryptochromes were characterized by subtracting genes of other subfamilies from 1309 candidate genes, and these features may interfere with the prediction of cryptochromes. However, most of the plant- and animal-type cryptochromes were captured by our pipeline. For example, the animal-type cryptochrome, TREU927 (*Trypanosoma brucei*), was captured even though the IPR005101 domain (DNA photolyase, FAD-binding/cryptochrome, C-terminal) was split into three subdomains because of nonconservative sequences insertions in the domain. In addition, five genes that encode the photolyase/cryptochrome family were captured and assigned accurately into four corresponding families, as reported in a previous study (17). Thus, our pipeline is able to accurately identify photolyase/cryptochrome families. Furthermore, our pipeline can detect fungal cryptochromes as well as plant- and animal-type cryptochromes, which demonstrates the comprehensive coverage of our method. For example, a DASH-type cryptochrome in the model fungus *Neurospora crassa* (18) was identified and classified into the CRY-DASH subfamily. In addition, PHR1 in *Trichoderma atroviridae*, which belongs to CPD photolyase class I (19), was also correctly identified in dbCRY.

**Figure 2.**
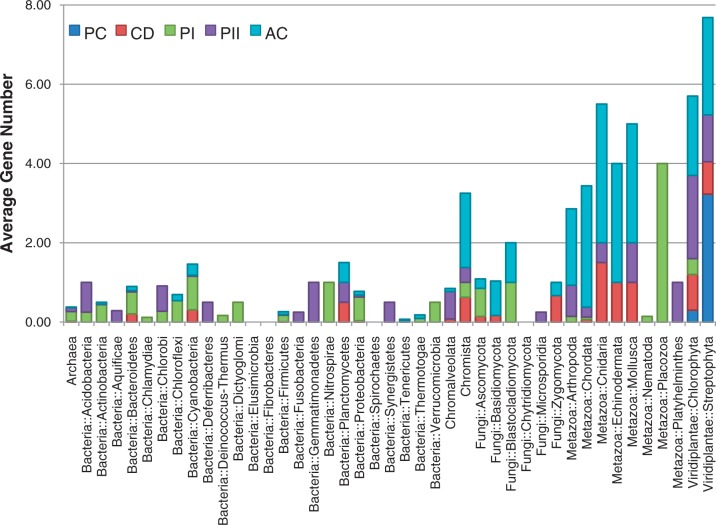
The taxonomic distribution of the number of genes encoding the five cryptochrome subfamilies. The distribution of the 1306 identified cryptochromes is shown in the accumulated chart. The average number of genes in each of the five subfamilies (PC, plant-type cryptochrome; CD, CRY-DASH; PI, CPD photolyase class I; PII, CPD photolyase class II; AC, animal-type cryptochromes including 6–4 photolyase) is shown for each taxonomy.

In total, 818 genomes have at least one of the putative genes from the photolyase/cryptochrome family, including genomes from 15 archaea, 561 bacteria, 150 fungi, 33 animals and 30 plants (Figure 2). Genes that belongs to CPD photolyase class I were the most frequently found; 621 genes were found in 590 genomes. Among these 590 genomes, 449 and 121 genomes belonged to bacteria and fungi, respectively, which indicates that the genomes of archaea, plants and animals rarely possess this type of blue-light regulator. The 74 plant-type cryptochromes were found only in the species belonging to the kingdom Plantae, with an exception in the subphylum Prasinophyceae. The number of genes encoding plant-type cryptochromes ranged from one to nine because of the varying number of alternatively spliced isoforms. In *Oryza sativa*, for example, six isoform products were captured among nine predicted plant-type cryptochromes. The genes encoding CRY-DASH proteins were distributed across a wide range of taxonomy, which is in accordance with previous research (20, 21). Unlike other classes, a low level of variance was observed in the number of CRY-DASH genes, which suggests that these genes do not have many alternative splicing isoforms. The genes encoding CPD photolyase class II were also found in a broad spectrum of taxa including bacteria, archaea, fungi, animals and plants but found in only 121 genomes. The genes encoding animal-type cryptochromes were also found in a broad spectrum of the taxa. In addition, high variance was detected in the phylum ‘Craniata’, ranging from 1 to 17, as shown in the high variance in plant-type cryptochromes (Figure 2).

## Utility of dbCRY

### Web interfaces and functionalities

To provide intuitive browsing of the identified genes, Web pages for visualizing charts and taxonomic distributions were developed. The distribution of different classes in each species and the distribution of different species for a given class can be visualized via the Web user interface. The taxonomic summary tables provide kingdom- and subphylum-wide distributions of the putative genes of the photolyase/cryptochrome family to offer a glimpse of the geographical landscape of those genes. In addition, the full list that describes the number of genes in each class from each of genome can be downloaded as a.sv file. A user can also browse the data by the five cryptochrome/photolyase subfamilies. In the ‘Browse by Class’ menu, the taxonomic distribution of the number of genes is provided as a box plot, which provides a quick overview of each class along with the wide range of taxonomy.

### Cross-linking favorite, a personalized virtual folder and data analysis hub

**Figure 3.**
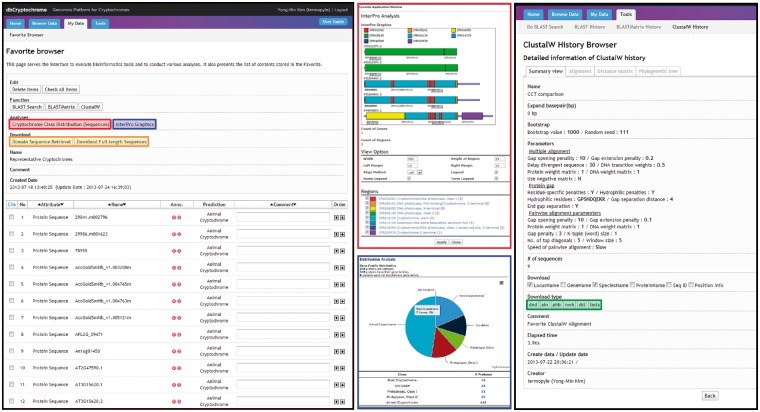
A screenshot of the Favorite Browser in dbCRY. Users are able to collect genes and investigate their domain architectures (red box) or composition of the genes of interest (blue box). The sequences can be downloaded with options of a full-length or a specific domain based on InterPro accessions (orange box). Users can also conduct DNA/protein alignments or phylogenetic analyses, and these results can also be downloaded.

dbCRY provides various functions via Favorite, a personalized data repository and analysis hub featured by the Comparative Fungal Genomics Platform (CFGP 2.0, http://cfgp.snu.ac.kr/) (22). dbCRY provides (i) class distribution, (ii) protein domain browser, (iii) domain sequence retrieval, (iv) homology search tools (BLAST and BLASTMatrix) and (v) a multiple sequence alignment tool (ClustalW; Figure 3). In particular, the domain sequence retrieval and multiple sequence alignment tools facilitate the search for conserved residues in the specific domains of selected cryptochromes. The Favorites created in dbCRY can be used in CFGP 2.0, which offers flexible and extended analysis options for further analysis. In addition, the Favorites are also interconnected with a number of gene family databases to offer multigene family analysis opportunities.

## System Structure

dbCRY was developed by taking the advantage of a three-tiered system, divided into the database, identification pipeline and Web interfaces. Genome and proteome data subjected to domain profiling were retrieved from the standardized genome warehouse of CFGP 2.0 (22). In addition to the InterPro domain information, additional results generated by seven bioinformatics programs were imported from CFGP 2.0 to provide broader information for each identified gene. The Web interface for dbCRY was based on the Data-driven User Interface of CFGP 2.0 to provide user-friendly environment. MySQL 5.0.81 (source code distribution) and PHP 5.2.6 were used for administrating database and developing Web interfaces, respectively. Web pages were served through Apache 2.2.9 Web server.

## Discussion

### Evolution of cryptochromes

Cryptochromes play a crucial role in the regulation of circadian rhythms. Cryptochromes have been identified in bacteria, animals and plants. Cryptochromes are related to DNA photolyases, which play a role in DNA repair. Although cryptochromes are structurally similar to DNA photolyase, they do not exhibit photolyase activity. Initially, bacterial DNA photolyase was speculated as the common ancestor of cryptochromes (10), but bacterial cryptochromes have not been characterized for decades. The discovery of cryptochromes in bacterial genome such as cyanobacterium *Synechocystis* sp. and *Vibrio cholera* and the discovery of CRY-DASH proteins from bacteria, plants, insects and vertebrates indicated that cryptochromes evolved before the origin of eukaryotic organisms (20, 23, 24). Furthermore, an early phylogenetic tree analysis suggested that animal- and plant-type cryptochromes diverged during two independent evolutionary events soon after the divergence of plants and animals (10). Thus, the discovery of bacterial cryptochromes and CRY-DASH proteins provides a new chance to investigate the evolutionary history and functional study of cryptochromes.

**Figure 4.**
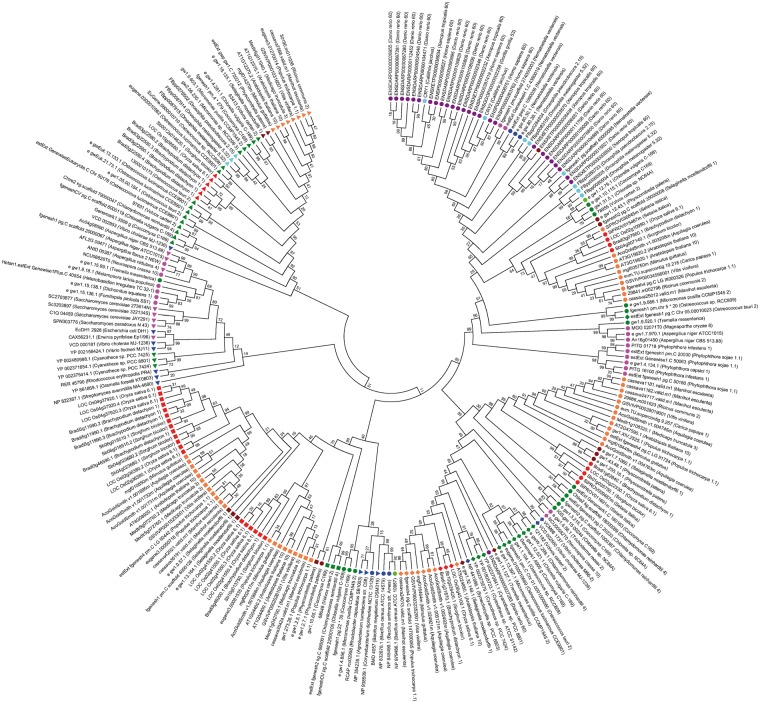
A phylogenetic tree of representative cryptochromes. The evolutionary relationships of 248 representative cryptochromes from 72 genomes were investigated. The evolutionary history was inferred using the minimum evolution method. The optimal tree with a sum of branch length = 49.91770009 is shown. Animal-type (circle), CPD photolyase class I (left handed triangle), CPD photolyase II (right handed triangle), CRY-DASH (diamonds) and plant-type (rectangular) cryptochromes formed distinct clusters. Different colors were used for indicating different taxa; violet (vertebrate), bright blue (insect), blue (bacteria), green (green algae), brown (moss), pink (fungi), red (monocot) and orange (eudicot).

In this study, 1309 cryptochromes-encoding genes were identified, and 248 cryptochromes from 72 genomes were selected and analyzed to investigate the evolution of cryptochromes. The phylogenetic tree of representative cryptochromes indicates that each cryptochrome subfamily forms a distinctive cluster (Figure 4). However, some animal-type cryptochromes from fungi and bacteria were detected in the CPD photolyase class I subfamily. Previous studies on the fusion proteins of 6–4 DNA photolyase suggest that evolutionary changes in the protein functionally separated the cryptochromes, but the core domain of photolyases were not significantly altered (25, 26). This mixed clade indicates that animal-type cryptochromes, including the 6–4 photolyases of fungi, bacteria and algae, have a higher sequence similarity to CPD photolyase class I compared with higher plants or vertebrates. Furthermore, the amino acid sequences between 6–4 DNA photolyase in plant- and animal-type cryptochromes showed high similarity, hampering clear distinction among them by domain profiles. The phylogenetic tree analysis showed that 6–4 DNA photolyase formed subclade in animal-type cryptochromes (Supplementary Figure S1). Thus, 6–4 DNA photolyase were included in animal-type cryptochrome in this study.

In contrast to photolyases, cryptochromes have a CCE region but poorly conserved structure (15). However, CCE domains of plants are more conserved compared with those of animals and also contain a DAS domain. The InterPro domain and phylogenetic analysis indicated that proteins containing the CCE domain were first detected in moss (*Physcomitrella patens*) and were not detected in green algae. These results agree with the previous research (1). In this study, 10 green algae genomes were investigated, but none included cryptochromes with the DAS domain. Overall, this result implies that the CCE domain may have emerged after the appearance of multicellular organisms and land plants. In contrast to green algae or aquatic plants, land plants evolved to adapt to various environmental conditions, including exposure to a broad spectrum of light. As a result, cryptochrome containing the CCE domain emerged to mediate various light responses. Genetic studies indicate that the DAS domain is important for intermolecular interaction, cellular localization and physiological function (27). The detection of DAS-containing CCE domains in moss suggests that land plants required the domain to mediate more complex responses to adapt to various light conditions. More specifically, CCE domains may have evolved to mediate new blue-light signaling pathways by interacting with their partners. However, cryptochromes containing the CCE domain were not observed in another moss species (*Selaginella martensii*). The origin, emergence and succession of DAS-containing CCE domains therefore remain to be elucidated. The identification of the evolution and origin of CCE domains will provide an important clue for evolutionary studies of cryptochromes.

In addition, the phylogenetic tree showed that cryptochrome 1 and 2 formed a distinct subclade (Figure 4). To investigate the divergence time of cryptochrome 1 and 2 in plant, phylogenetic analysis were performed using 144 plant-type cryptochromes including cryptochromes of *Amborella trichopoda*. The phylogenetic tree showed that cryptochrome 1 and 2 were subdivided before the divergence of monocots and eudicots. These two cryptochromes therefore evolved in a lineage-specific manner (Supplementary Figure S2). In conclusion, these data suggest that the evolution of cryptochromes resulted from an adaptation to various light conditions as land plants were exposed to more complex and various environments.

### Application of dbCRY

Cryptochromes are structurally related to DNA photolyases but do not possess DNA repair activity. The crystal structure of plant and bacterial cryptochromes reveals the structural differences between cryptochromes and photolyases (20, 28), and functionally essential amino acids that cause catalytic differences between these proteins have been identified. Although cryptochromes are structurally different from photolyases, the mode of action of cryptochromes has been revealed by comparative analysis between cryptochromes and photolyases. For example, a comparative analysis of photolyases and cryptochromes has identified the essential amino acids for a light-activated electron transfer reaction (13). Thus, a comparative analysis between plant- and animal-type cryptochromes may unveil the function of cryptochromes and their evolution.

In comparative studies, site-directed mutagenesis of conserved amino acids, deletion of specific domains or domain-swapping experiments based on the sequence comparison among cryptochromes are commonly used. The comparison of large numbers of cryptochrome sequences from various genomes may allow bench scientists to discover important amino acids or domains in cryptochromes. However, the large volume and disorganization of genome information is often a barrier to these comparisons. It is difficult to identify individual gene families from a wide range of genomes because of complicated structures or divergent domain architectures. In addition, special skills are required to handle the information derived from sequence alignments or the parsing of specific domain sequences. dbCRY can provide accurate and various sequence information and tools for analyzing cryptochromes. By using this database, researchers can perform sequence similarity search (BLAST) with putative cryptochrome sequences to evaluate and determine conserved amino acids by the alignment of specific domains or full-length sequences using CLUSTALW (Figure 3). The embedded phylogenetic analysis tool in dbCRY allows users to analyze either domain sequences of interest or full-length cryptochromes. In addition, dbCRY provides taxonomy information for each cryptochrome, and the user can remove or add cryptochrome sequences to their Favorites. Therefore, dbCRY enables bench scientists to collect functionally related proteins or cryptochromes of specific taxonomy for comparative analysis.

In conclusion, the genome-wide identification and analyses of cryptochromes, such as the discovery of CRY-DASH or the characterization of bacterial cryptochromes, have unveiled the evolution of cryptochromes as well as their biological function. With accumulation of additional information, further genome-wide studies will be able to uncover the old branch or the origin of cryptochromes.

## Future Directions

We believe that dbCRY is the most comprehensive database for genes encoding blue-light photoreceptors. This database has been developed to support bench scientists who study the mechanisms and biochemical properties of cryptochromes. The extensive taxonomy that is archived in dbCRY will assist researchers in many ways, including the collection of candidate cryptochrome targets, the determination of conserved residues for mutagenesis and comparative and evolutionary studies among genes that encode cryptochrome. To keep up with the up-to-date and broader range of genome information, the dbCRY will be updated on the regular basis when new or updated genomes in major repositories, such as NCBI and Ensembl, become available. We will continue our efforts to provide additional useful tools to make the dbCRY a better research portal for cryptochrome genomics.

## Availability

All the data and functions described in this study can be freely accessed at the dbCRY Web site (http://www.dbcryptochrome.org/).

## Supplementary Data

Supplementary Data are available at *Database* Online.
